# Development of a filter to prevent infections with spore-forming bacteria in injecting drug users

**DOI:** 10.1186/s12954-016-0122-1

**Published:** 2016-12-01

**Authors:** Nour Alhusein, Jenny Scott, Barbara Kasprzyk-Hordern, Albert Bolhuis

**Affiliations:** 1 Department of Pharmacy and Pharmacology, Claverton Down, Bath, BA2 7AY UK; 2 Department of Chemistry, University of Bath, Claverton Down, Bath, BA2 7AY UK

**Keywords:** Filter, PWIDs, Heroin, Bacterial spores, Anthrax

## Abstract

**Background:**

In heroin injectors, there have been a number of outbreaks caused by spore-forming bacteria, causing serious infections such as anthrax or botulism. These are, most likely, caused by injecting contaminated heroin, and our aim was to develop a filter that efficiently removes these bacteria and is also likely to be acceptable for use by people who inject drugs (i.e. quick, simple and not spoil the hit).

**Methods:**

A prototype filter was designed and different filter membranes were tested to assess the volume of liquid retained, filtration time and efficiency of the filter at removing bacterial spores. Binding of active ingredients of heroin to different types of membrane filters was determined using a highly sensitive analytical chemistry technique.

**Results:**

Heroin samples that were tested contained up to 580 bacteria per gramme, with the majority being *Bacillus* spp., which are spore-forming soil bacteria. To remove these bacteria, a prototype filter was designed to fit insulin-type syringes, which are commonly used by people who inject drugs (PWIDs). Efficient filtration of heroin samples was achieved by combining a prefilter to remove particles and a 0.22 μm filter to remove bacterial spores. The most suitable membrane was polyethersulfone (PES). This membrane had the shortest filtration time while efficiently removing bacterial spores. No or negligible amounts of active ingredients in heroin were retained by the PES membrane.

**Conclusions:**

This study successfully produced a prototype filter designed to filter bacterial spores from heroin samples. Scaled up production could produce an effective harm reduction tool, especially during outbreaks such as occurred in Europe in 2009/10 and 2012.

**Electronic supplementary material:**

The online version of this article (doi:10.1186/s12954-016-0122-1) contains supplementary material, which is available to authorized users.

## Background

In Europe, there are around 1.3 million problem opioid users, the majority of whom inject [1]. People who inject drugs (PWIDs) frequently suffer from infections, in particular, skin and soft tissue infections, which are estimated to cost the NHS in the UK up to £30 million annually [2]. Since 2000, there have been, across Europe, several outbreaks amongst PWIDs of particularly serious, life threatening infections caused by brown heroin that is contaminated with bacteria such as *Bacillus* and *Clostridium*, leading to severe skin and soft tissue infections such as anthrax, wound botulism, gas gangrene and tetanus [3]. In 2000, there were 60 confirmed cases of *Clostridium novyi* infection amongst PWIDs in Scotland, with an 87% fatality rate [4], with further cases reported across Europe. In 2009/10, there was a notable outbreak with 119 cases of injectional anthrax; 19 deaths were reported in the UK and Germany. Since then, further cases and mortalities have been reported from Germany, Denmark, UK and France [5]. Harm reduction response during such outbreaks is at present limited, with advice given usually limited to ‘switch to smoking, do not inject’ [4]. An alternative solution could be to use filters, but there are no filters available for heroin users that are easy to use and which can remove bacteria (see also below). Driven by the realisation of the health research community that no real solutions are provided to deal with outbreaks of infections by spore-forming bacteria, we decided to develop a filter that could be distributed for use by PWIDs to prevent infections and meet PWIDs’ acceptance criteria.

*Bacillus* and *Clostridium* species are commonly found in soil, dust and human/animal faeces and are thought to contaminate brown (base) heroin during production, storage and transportation [6]. Characteristic of both bacilli and clostridia is that, in the absence of nutrients, they form dormant spores that are highly resistant to stresses such as high temperatures, desiccation, UV irradiation and chemical damage. As a consequence, bacterial spores survive for long periods of time and only germinate and develop into actively growing bacteria again when conditions become favourable. One such ‘favourable’ condition is when spores are injected intravenously, intramuscularly or subcutaneously.

Many PWIDs filter their heroin before injection, but this only removes particulates to prevent needle blockage and, most likely, reduces small blood vessel damage. Often, PWIDs use homemade pieces of material from cotton wool or cigarettes; these are not sterile and could be a further source of contamination [7]. Some needle and syringe programmes supply filters, but even those commercially available only remove particles (solid materials found in brown heroin, e.g. from poppy straw) that are larger than bacterial spores. The exclusion limit of these filters is, at best, ~10 μm [8], whereas the average diameter of *Bacillus anthracis* (the causative agent of anthrax) spores is ~0.8 μm [9]. Whether PWIDs use supplied filters or homemade items, bacterial spores are thus not removed and, if present, could lead to serious and potentially lethal infections. The aforementioned wheel filters are available with a pore size of 0.2 μm and can thus remove bacterial spores, but these only fit syringes with detachable needles and cannot be used with commonly used fixed needle syringes. In addition, these wheel filters retain a significant amount of drug [10] thus reducing the effect of the drug. Therefore, not all PWIDs would find the use of such filters acceptable.

As mentioned above, the main cause of infections with *Bacillus* and *Clostridium* species is contaminated heroin [3–5, 11]. The ‘street’ process for preparing brown heroin includes the use of acidic substrates such as citric acid in water and flame heating [7]. These processes do not destroy bacterial spores [12], and effective filtration of heroin after acidification and heating could be a viable option to remove spores. However, membranes with pores small enough to remove bacterial spores are easily blocked with particulates. Heroin that is prepared as above still contains a lot of particulates which will block membranes with small pores, making these not acceptable for heroin users. Our aim was therefore to develop a filtration device in which a prefilter removes particulates, followed by a 0.2 μm filter to remove bacterial spores. This device has to remove particulates and bacterial spores efficiently and be also acceptable to users. Thus, it should not block due to particulates, retain no or negligible volume of injection (or it would reduce the hit so be unacceptable), have a fast filtration time and also have minimal binding of the active ingredients in heroin to the filter (again so as not to impact on effect). In addition, it has to be easy to use. Unless the filter device possesses all these qualities, it may not be acceptable to PWIDs to incorporate such a device into their drug preparation rituals.

## Methods

### Bioburden testing

To get an indication of the microbial contamination in samples of heroin we obtained from the local police in Bristol from several police seizures across the Avon and Somerset constabulary area, the bioburden was determined using a plate-based method. In brief, 100 mg samples of heroin were suspended in 1.5 mL phosphate buffered saline (PBS), vortexed for 15 min and further diluted tenfold. Fifty microlitres was plated on brain heart infusion (BHI) agar (Oxoid) or Sabouraud agar (Oxoid). To detect bacteria, the BHI agar plates were incubated aerobically at 37 °C for 24 h or anaerobically in a GasPak container (Becton Dickinson) at 37 °C for up to 10 days. To detect fungi, Sabouraud agar plates were incubated aerobically at 28 °C for 7 days. To identify microbial species, the 16 ribosomal RNA gene from a number of colonies was amplified by the polymerase chain reaction (PCR), using OneTaq polymerase (New England Biolabs) and one of three oligonucleotide pairs (for sequences of these oligonucleotides, see Additional file 1 and References [13–15]). The bacterial species were identified by sequencing the amplified products and comparing these to the NCBI nucleotide database (www.ncbi.nlm.nih.gov/nucleotide).

### Heroin sample preparation

Heroin samples were prepared, with some minor modifications, following the method described, which is a standard method for preparing brown heroin in Europe [7, 10]. One hundred milligrams of brown heroin and 50 mg citric acid (Exchange Supplies Ltd.) was added to 0.7 mL water that was produced using a Milli-Q water purification system (Millipore). This was heated in a Stericup® (Apothicom, distributed by Exchange Supplies Ltd.) until it just started to boil and became clear. The volume of the solution was measured using a 1-mL insulin-type syringe (Unisharp 1-mL fixed needle syringe; Exchange Supplies Ltd.), and to correct for evaporation, distilled water was added to make the total volume of the sample 0.8 mL.

### Filtration devices and membranes

Filtration of samples was performed with the following filter devices: 25 mm Minisart RC25 syringe filters (0.2-μm pore size, Sartorius), 15 mm Minisart RC15 (0.45-μm pore size, Sartorius), 13 mm Millex GV (0.22-μm pore size, Millipore) and 4 mm Millex GV (0.22-μm pore size, Millipore) and a Swinnex filter holder (fitted with a 13 mm membrane, Millipore). For the latter, we tested all suitable types of membrane that were available with the correct diameter and pore size (13-mm diameter, 0.22-μm pore size, all from Millipore): hydrophilic polyvinylidene fluoride (PVDF), polytetrafluorethylene (PTFE), mixed cellulose ester (MCE), polyethersulfone (PES) and hydrophilic nylon. Where indicated, membranes were combined with a glass fibre prefilter (13 mm in diameter, 2-μm pore size).

### Determination of retention volume

To determine the retention (hold-up) volume of filtration devices, heroin samples were prepared as above, and the volume of the solution was measured using a 1-mL insulin-type syringe. After filtration, the volume was measured again with a 1-mL syringe to determine the loss in volume.

### Binding of active ingredients in heroin to membranes

Heroin samples were prepared as above and the volume of the solution was adjusted to 2 mL with distilled water. The volume used was more than in a normal heroin preparation, which could have increased solubility of active ingredients, but the main aim here was to analyse the effects on concentrations of active ingredients before and after filtration through different types of membranes. This was done by dividing each sample into two aliquots; 1 mL was filtered and the other 1 mL was kept unfiltered. Filtration was performed with Swinnex filter holders and membranes listed above. In each case, the assembly consisted of a prefilter with membrane, except when the prefilter was tested on its own. Heroin samples were diluted with distilled water/methanol (85:15 *v*/*v*) to a final concentration of 800 ng/mL with the addition of an internal standard (morphine-d3, 6-monoacetylmorphine-d3 and heroin-d9 at a concentration level of 10 ng/mL). Heroin in samples was then quantified by liquid chromatography coupled with tandem mass spectrometry) LC-MS/MS using a Waters Acquity UPLC system and Waters Xevo TQD triple quadrupole mass spectrometer with a Chiralpak CBH HPLC column (5-μm particle size, 10 cm × 2.0 mm; Chiral Technologies, France) as described [16].

### Filtration of heroin samples with *B. subtilis* 168 spores

Bacterial spores of *B. subtilis* 168 [17] were harvested by first growing overnight in Luria broth (LB; 1% tryptone, 0.5% yeast extract, 1% NaCl), diluting 100-fold in 50 mL Schaeffer sporulation medium [18] and further growth in a shaking incubator at 37 °C for 3 days. Next, the spores were collected by centrifugation (5000*g*, 20 min), re-suspended in 10 mL water and centrifuged again, re-suspended in PBS and stored at 4 °C.

Heroin samples were prepared as above and *B. subtilis* spores were added to a final concentration of ~10^8^ spores/mL. These samples were filtrated, and the flow-through was plated on LB agar plates for enumeration.

### Manufacturing of filtration device

The filtration device was made from machine-grade polycarbonate (Durbin Metal Industries), manufactured using standard machine tools. The two main parts (syringe holder and the filter collar) were screwed together. A butyl rubber O-ring was used around the syringe port to prevent the air leakage (the O-ring).

## Results

### Bioburden testing

A critical factor in sterilisation of products such as drugs or food is the bioburden, i.e. the number of microbes contaminating the product. The higher the bioburden, the greater the chance that some viable microbes are still present after a sterilisation process. It is thus important to know the bioburden before any process of removing or killing microbes is employed. A number of studies have shown that 90–95% of heroin samples are contaminated with bacteria (mainly bacilli) without reporting the bioburden (see [6]), while only one study reported bioburdens, which were found in the range of 1.6 × 10^2^–3.7 × 10^4^ organisms per gramme heroin [19]. There may, however, be significant differences depending on the origin of the heroin, and we therefore tested the bioburden of the heroin samples we obtained. Samples were plated on agar plates and then incubated to detect bacteria (BHI agar plates) or fungi (Sabouraud agar). From this, we found that the heroin contained up to 580 bacteria per gramme of heroin. Obligate anaerobic organisms, which includes *Clostridium* spp., were not found, and also, no fungal contaminants were isolated.

Gram staining of 20 bacterial colonies from the BHI agar plates showed that all were Gram-positive, with one being coccoid and the remainder rod-shaped. From these, 11 colonies were identified to the species level, using the commonly used method of sequencing of the 16rRNA gene and comparing this to known sequences in a nucleotide database. The rod-shaped bacteria were all identified as *Bacillus* species: three colonies were identified as *B. licheniformis*, two as *B. pumilus*, two as *B. subtilis*, one as *B. thermolactis* and one as *B. massiliosenegalensis*. The coccoid organism isolated was identified as *Staphylococcus hominis*.

### Testing existing filtration devices

In order to remove bacteria (including spores) from heroin, filtration would be the only method that is practical, as the heating of heroin during its preparation does not kill bacterial spores (see below and Ref [12]). PWIDs would not want drug losses due to filtration; if a significant amount of drug remained in the filter, users would be tempted to extract drug from those filters. A case in point is the use of cotton pellets by heroin users, which in some cases are handled with unwashed hands, stored and re-used to recover any remaining heroin [10]. That is an unsafe practise, as storage of wet filters can lead to growth of microbes and increase risks of infections.

**Fig. 1:**
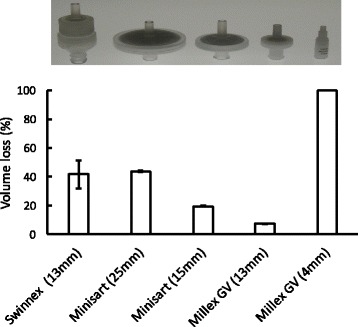
Evaluating the loss of volume in a 0.8 mL heroin sample by filtration using commercially available syringe filters. The Swinnex filter holder was fitted with a 13 mm PVDF membrane. The top panel shows an image of the filters used, with the corresponding data on the loss of volume from these filters in the graph below

We first tested loss of filtration of heroin samples using existing syringe filters that are available for laboratory research. This included standard syringe filters with different diameters (4 mm, 13 mm, 15 mm and 25 mm) and a Swinnex filter housing which allows for assembly of the filter casing with a 13 mm membrane of choice. In case of the filters with membrane diameters of 13-25 mm, losses ranged from 7.5% (13 mm Millex GV filter) to 44% (Minisart RC25 (25 mm) filter (Fig. 1). In these cases, the losses were due to some of the heroin being left in the filter housing, thus representing the retention (hold-up) volume. In case of the 4 mm Millex GV filter, it was not possible to filter the sample as the filter immediately blocked up due to the presence of particulates in the heroin. It should be noted that other brands of syringe filters are available (in particular in the range of 25–30 mm), but we did not test differences between brands.

### Effect of membrane filters on active ingredients in heroin

**Fig. 2:**
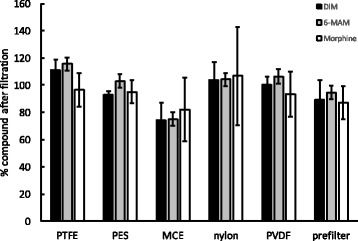
The percentage of active ingredients after filtration using different membrane filters (*n* = 3). For all ingredients, the amount before filtration was normalised to 100%

In addition to minimising losses due to the retention volume, it is also important that the active ingredients in heroin are not lost by binding to the membrane. To analyse this, we firstly determined the main active ingredients in the heroin samples, using a highly sensitive analytical chemistry (LC-MS/MS) technique. The main active ingredients were diamorphine (DIM 82.2%) and 6-monoacetylmorphine (6-MAM 17.6%), with a small amount of morphine being present (0.2%). Next, we determined the amount of these ingredients before and after filtration through six types of membranes (PTFE, PES, MCE, nylon, PVDF and glass fibre prefilter) that were assembled in a Swinnex filtration device. Filtration with most of the membranes showed little or no loss of active ingredients (Fig. 2). An exception was the MCE, which exhibited an average loss of ~25% of the active ingredients. Removal of active ingredients would not be acceptable for PWIDs, and MCE is therefore not suitable for filtration of heroin.

### Design of a novel filter

In the design of a filter for PWIDs, there were two important requirements: the loss of drug by filtration should be low, and the device should be easy to use. As shown above, the Millex GV filter, with a 13 mm membrane, had the lowest retention volume. It would be expected that this volume would be less with a 4 mm membrane, but this was unusable as the filter blocked up immediately. This blocking up could be prevented by filtrating with a 2 μm glass fibre prefilter before filtration through the bacterial filter. This was achieved by cutting a prefilter to the required size and adding this to a 4 mm syringe filter. The retention volume was not determined in this case, as cutting the prefilter was not very accurate, leading to variability in the effectiveness of the filter. Nevertheless, it does show that removing larger particles before filtration through a 0.2 μm membrane improves the flow properties.

On the second requirement, ease-of-use, syringe filters do not perform particularly well. Standard syringe filters are normally driven by positive pressure, i.e. a solution is pushed through the filter with the aid of a syringe. The filtrate needs to be collected in a sterile container and then drawn up in a second sterile syringe before it is ready for injection. A faster alternative method is to draw the heroin into the syringe through a filter, using the negative pressure generated by the syringe, which reduces the number of steps. However, the syringe filters that we tested, as well as most other syringe filters, are not designed to work in reverse flow. And whichever direction of flow is used, standard syringe filters do not fit syringes with a fixed needle, which are commonly used by PWIDs

**Fig. 3:**
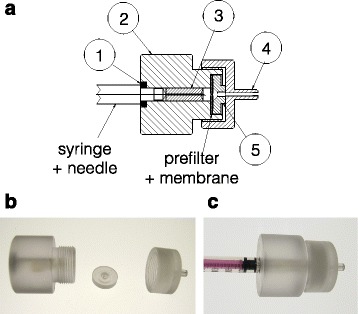
Filtration device. **a** Schematic overview of the prototype filtration device, which is assembled from five parts: (*1*) butyl rubber O-ring, (*2*) syringe holder, (*3*) needle collar, (*4*) filter inlet and (*5*) membrane plate. The prefilter and membrane are two separate parts that are pressed together by the membrane plate. **b** Image of the three main components of the filtration device; from left to right the syringe holder, membrane plate and filter collar. **c** Image of filtration device with a fixed needle syringe

Taking the above in consideration, we had two main requirements in the design of a prototype filter. Firstly, it should have good flow properties so that it can be used in reverse flow, and secondly, the filtration device should be able to accommodate syringes with a fixed needle to minimise the number of steps required for injecting drugs. A prototype device was made from polycarbonate (Fig. 3), using machine tooling in the local workshop at the University of Bath. The two main parts are the syringe holder (part 2) and filter inlet (part 4), which fit together with a screw thread. The syringe (with fixed needle) fits in the syringe holder with the needle slotted in the needle collar (part 3). To ensure an air-tight fit of the syringe in the device, a butyl rubber O-ring was fitted (part 1) as indicated. To prevent blocking of filter by particulates, a 2 μm glass fibre prefilter was combined with a 0.2 μm filter in the device (both 13-mm diameter). The prefilter and membrane are held together in a fixed position by the membrane plate (part 5).

### Filtration time of the prototype filtration device

**Fig. 4:**
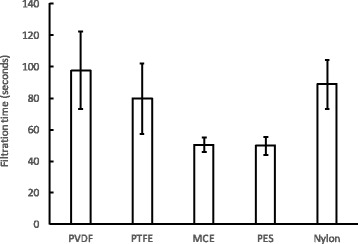
Filtration time of 0.7 mL of heroin using different membranes. All membranes were combined with a prefilter in the prototype filtration device

We assembled inside the prototype filter the glass microfibre prefilter with different 0.2 μm membrane filters to test which combination showed the best flow properties. The filters tested were PVDF, PTFE, MCE, PES and nylon. With those, we tested the time it takes to filtrate a typical heroin sample of 0.7 mL. This measure is not necessarily an indication of the most efficient filtration of heroin, but an overly long filtration process would not be acceptable to PWIDs. The filtration time of heroin using a small cotton pellet that is provided with a Stericup® pack that the drug users frequently use is around 30 s. Our data showed that filtration through MCE and PES membranes was the fastest, taking around 50 s (Fig. 4). This was slower than filtration through the cotton pellet, but that is not surprising considering the difference in pore size. Filtration through the other membranes was even slower, with filtration times up to twice as long, indicating that physical and chemical characteristics of the polymer used in the membrane also play an important role in the rate of flow. As our requirement was fast flow and low binding of active ingredients, further tests were performed with PES membranes only.

### Removal of bacterial spores using the prototype filtration device

We tested the capability of removing bacterial spores from heroin with the prototype filtration device and a PES membrane. To this purpose, a 0.8 mL heroin sample was spiked with *B. subtilis* spores (~10^8^ spores), followed by filtration. After this, samples were tested for contamination with *B. subtilis* spores by plating on agar plates and incubation at 37 °C. No growth was observed after filtration, demonstrating that the filtration device removed bacterial spores efficiently.

During preparation, heroin is heated in the presence of acid to aid dissolution. Directly after preparation, we measured the temperature to be 70 ± 5 °C (*n* = 3), which cools down to 30 ± 2 °C in 4 min. We tested whether filtration of heroin that was still hot would affect filtration, e.g. by damaging the membrane or enlarging the pore size due to the increased temperature. To test this, heroin samples spiked with *B. subtilis* spores were heated to 75 °C and then filtrated either immediately or after 10 min of cooling. In both cases, no spores passed through the filter device, showing that temperature of the heroin does not affect the performance of the PES membrane.

Notably, heating in the presence of acid and heroin does not affect viability of *B. subtilis* spores to a great extent; we observed a survival of spores of 57% (+/−8%) after heating *B. subtilis* spores in the standard heroin preparation. These survival rates may not be the same for spores from other bacteria, but a previous study also showed that *C. novyi* spores survive the heroin preparation process [12].

### Reusability

The prototype filter was tested as well to find out whether it can be re-used, as this is something that should be discouraged due to sharing risks. After using the filter once, a second heroin sample was prepared, and the same filter was used again. We found that the filtration process was not efficient; out of 770 μL heroin sample before filtration, only 100 μL passed through the filter into the syringe. This is due to a layer of particles (filter bed) that formed on the filter membrane during the first filtration process, thus leading to blockage of the device for further use. Thus, the prototype would discourage sharing and reuse. It should be noted that there may be variation in batches of heroin and preparation procedures by PWIDs; reusability with such varying conditions was not tested by us.

## Discussion

Heroin contaminated with bacteria may lead to outbreaks of infectious diseases. The majority of the bacteria identified in brown heroin that we obtained from the local police were *Bacillus* spp., which are sporulating bacteria that are normally found in soil. It is not surprising that mainly sporulating bacteria were found, as production of heroin from opium involves several steps that require heating and treatment with various chemicals [20], and only the very resistant bacterial spores could survive these chemicals. None of the bacteria we identified were known to cause disease although it cannot be excluded that they would become pathogenic when injected. Nevertheless, it seems quite likely that only occasional batches with heroin are contaminated with true pathogens such as *B. anthracis* or *Clostridium* species. One colony was identified as *S. hominis*; this is a non-sporulating bacterium that is commonly found on skin and it probably contaminated the heroin after production, e.g. during packaging.

The number of bacteria found in the heroin that we obtained was ~580 bacteria per gramme, which is within the range that was published before [19]. This does not seem a particularly high number, but it should be noted that only a few spores are needed to cause an anthrax infection [21]. Additionally, many PWIDs have poor physical health, which may increase the probability of getting an infection.

Current options for PWIDs to filter heroin include the use of homemade filters from cigarette filters or cotton pellets or purpose-made filters provided by pharmacies or harm reduction programmes (e.g. Sterifilt®, produced by Apothicom) that remove particulates from heroin [7, 8, 10]. These do not remove bacterial contamination [7, 8], but here, we show a prototype filtration device that we developed, a viable option for the removal of bacterial spores and particulates from heroin. Based on both flow properties and low binding of active ingredients of heroin, a prefilter combined with a PES membrane proved to be the most efficient combination. The prefilter removes particulates from the heroin, thereby preventing blocking of the 0.2 μm filter capable of removing bacterial spores. Effectiveness of removal of bacterial spores was tested with spores from *B. subtilis*, which is a non-pathogenic relative of *B. anthracis* (and is thus much safer to work with), with spores that are smaller than those of most other sporulating bacteria, including *B. anthracis* [9]. Thus, a filter designed to work with *B. subtilis* spores will thus also efficiently remove spores from most bacterial species.

A limitation of our study was that experiments were performed with heroin prepared in a standardised manner. However, there can be considerable variation between both batches of heroin (e.g. different cutting agents) and heroin users’ practise; such variations were not tested in our laboratory setting.

The prototype filtration device was designed in such a way that it would accommodate syringes with fixed needles, such as insulin-type syringes. That would allow for the preparation of heroin in a simple step by directly drawing up heroin through the filter into the syringe, similar to practises currently used by PWIDs who use products such as Sterifilt® filters [7]. Due to limitations of the tooling equipment used, the device we designed is rather bulky, but the prototype can be made significantly smaller and consisting of fewer parts when it is manufactured by moulding instead.

We demonstrated that the prototype filter is effectively ‘single use’ because it blocks on reuse, so it could potentially discourage reuse and sharing. This has advantages for PWIDs in terms of reducing health risks. We also showed that the prototype filter does not retain any significant amounts of drug for a standardised heroin sample, and therefore, there is no motive to save them for ‘bashing down’ at a later date when no drug or money is available. This feature may of course make it unacceptable to some PWIDs, who could find this a daunting prospect.

We envisage that after user acceptability testing and suitable production scale-up, our filter could form the cornerstone of infection prevention during anthrax or other spore-forming bacteria outbreak amongst PWID. It could be distributed through Needle and Syringe Programmes (NSPs) at the first detection of an outbreak or ideally supplied continuously. If used for every injection, it would make the use of sterile water (used by some PWIDs) less of a necessity, as any microbes and particles, e.g. from tap water, would also be removed during filtration.

## Conclusions

Infections in PWIDs are common, with one potential source of bacterial pathogens being the heroin itself. There have been several outbreaks of infections by spore-forming bacteria in PWIDs, resulting in high morbidity and mortality. It is thus important to test and develop filtration devices which can remove bacterial spores and prevent such outbreaks. In this paper, a filtration device was developed which removes bacteria and particulates, is potentially easy for PWIDs to use and does not collect active ingredients, thus does not reduce the ‘high’. A future version needs to be streamlined, as the prototype is too bulky and its manufacture would be expensive. Such an improved device could be provided to attract PWIDs to existing injecting equipment programmes (IEPs) across Europe and beyond, especially during an outbreak of anthrax or other spore-forming bacterial infections. The overall aim of our filter is to reduce the health impact of injecting drug use and save healthcare costs.

## Additional file

Additional file 1: Sequences of oligonucleotides used for sequencing 16 ribosomal RNA genes. (DOCX 41 kb)

## Abbreviations

6-MAM: 6-monoacetylmorphine
BHI: Brain heart infusion
DIM: Diamorphine
IEPs: Injecting equipment programmes
LC-MS/MS: Liquid chromatography tandem mass spectrometry
MCE: Mixed cellulose ester
PBS: Phosphate buffered saline
PES: Polyethersulfone
PTFE: Polytetrafluorethylene
PVDF: Polyvinylidene fluoride
PWIDs: People who inject drugs
