# Supplementary material for: Development of a filter to prevent infections with spore-forming bacteria in injecting drug users
Source: Harm Reduct J. 2016 Dec 1;13:33. doi: 10.1186/s12954-016-0122-1 (PMC5131546; doi:10.1186/s12954-016-0122-1)
Supplement: Additional file 1: — Sequences of oligonucleotides used for sequencing 16 ribosomal RNA genes. (DOCX 41 kb) [file 12954_2016_122_MOESM1_ESM.docx]

Primer sets used to amplify bacterial 16S RNA genes

| **Primer name** | **Sequence** | **Reference** |
| --- | --- | --- |
| 63f | CAGGCCTAACACATGCAAGTC | 10 |
| 1387r | GGGCGG(A/T)GTGTACAAGGC | 10 |
|  |  |  |
| BAK11w | AGTTTGATC(A/C)TGGCTCAG | 11 |
| BAK2 | GGACTAC(A/C/T)AGGGTATCTAAT | 11 |
|  |  |  |
| 8f | AGAGTTTGATCCTGGCTCAG | 12 |
| 1541r | AAGGAGGTGATCCAGCCGCA | 12 |
